# 22511 Glycolipid-loaded nanoparticles harness iNKT cells for tumor immunotherapy

**DOI:** 10.1017/cts.2021.406

**Published:** 2021-03-30

**Authors:** Travis Shute, Elizabeth Dudley, Andrew Lai, Briana Salas, Brandy Vincent, Daniel Angel, Kelly Nash, Elizabeth Leadbetter

**Affiliations:** 1 UT Health San Antonio; 2 UTSA

## Abstract

ABSTRACT IMPACT: My work is on the development of a novel tumor immunotherapy to treat various types of cancer OBJECTIVES/GOALS: As iNKT cells can have direct and indirect killing effects on tumor cells, we propose a novel strategy for activating iNKT cells, via a PLGA nanoparticle delivery platform, to promote anti-tumor immune responses. METHODS/STUDY POPULATION: Poly-lactic-co-glycolic acid (PLGA) nanoparticles can be reproducibly loaded with an iNKT cell glycolipid agonist, alpha-galactosylceramide (αGalCer), and a tumor associated antigen, ovalbumin (OVA). We then test our nanoP prophylactically and therapeutically against a murine model of melanoma, B16F10-OVA. RESULTS/ANTICIPATED RESULTS: These dual-loaded PLGA nanoparticles rapidly activate iNKT cells in vivo to produce IFNgamma. Furthermore, in an in vivo model of melanoma, using B16F10-OVA cells, both prophylactic and therapeutic administration of nanoparticles containing αGalCer and OVA led to decreased tumor cell growth and increased survival. We also show our nanoparticle therapy has synergistic potential with clinically used immune checkpoint blockade (ICB) therapies, anti-PD-1 and anti-CTLA-4, indicated by the significance increase in survival and lower tumor growth rate of ICB +nanoP treated mice compared to either ICB or nanoP alone. DISCUSSION/SIGNIFICANCE OF FINDINGS: This novel delivery system provides a platform with tremendous potential to harness iNKT cells for cancer immunotherapy purposes against many cancer types.

